# Robocasting and Laser Micromachining of Sol-Gel Derived 3D Silica/Gelatin/β-TCP Scaffolds for Bone Tissue Regeneration

**DOI:** 10.3390/gels8100634

**Published:** 2022-10-07

**Authors:** María V. Reyes-Peces, Eduardo Félix, Francisco J. Martínez-Vázquez, Rafael Fernández-Montesinos, Óscar Bomati-Miguel, María del Mar Mesa-Díaz, Rodrigo Alcántara, José Ignacio Vilches-Pérez, Mercedes Salido, Nicolás De la Rosa-Fox, Manuel Piñero

**Affiliations:** 1 Departamento de Física de la Materia Condensada, Facultad de Ciencias, Universidad de Cádiz, 11510 Puerto Real, Spain; 2 Institute of Research on Electron Microscopy and Materials (IMEYMAT), Universidad de Cádiz, 11510 Puerto Real, Spain; 3 Departamento de Ingeniería Mecánica, Energética y de los Materiales, Universidad de Extremadura, 06006 Badajoz, Spain; 4 Departamento de Histología, Facultad de Medicina, Universidad de Cádiz, 11004 Cádiz, Spain; 5 Instituto de Biomedicina de Cádiz, INIBICA, Universidad de Cádiz, 11009 Cádiz, Spain; 6 Departamento de Ingeniería Química, Facultad de Ciencias, Universidad de Cádiz, 11510 Puerto Real, Spain; 7 Departamento de Química-Física, Facultad de Ciencias, Universidad de Cádiz, 11510 Puerto Real, Spain

**Keywords:** 3D scaffold, hybrid, sol-gel ink, robocasting, laser micromachining, osteoblasts, regenerative medicine, bone tissue engineering, cytoskeleton, focal adhesion

## Abstract

The design and synthesis of sol-gel silica-based hybrid materials and composites offer significant benefits to obtain innovative biomaterials with controlled porosity at the nanostructure level for applications in bone tissue engineering. In this work, the combination of robocasting with sol-gel ink of suitable viscosity prepared by mixing tetraethoxysilane (TEOS), gelatin and β-tricalcium phosphate (β-TCP) allowed for the manufacture of 3D scaffolds consisting of a 3D square mesh of interpenetrating rods, with macropore size of 354.0 ± 17.0 μm, without the use of chemical additives at room temperature. The silica/gelatin/β-TCP system underwent irreversible gelation, and the resulting gels were also used to fabricate different 3D structures by means of an alternative scaffolding method, involving high-resolution laser micromachining by laser ablation. By this way, 3D scaffolds made of 2 mm thick rectangular prisms presenting a parallel macropore system drilled through the whole thickness and consisting of laser micromachined holes of 350.8 ± 16.6-micrometer diameter, whose centers were spaced 1312.0 ± 23.0 μm, were created. Both sol-gel based 3D scaffold configurations combined compressive strength in the range of 2–3 MPa and the biocompatibility of the hybrid material. In addition, the observed Si, Ca and P biodegradation provided a suitable microenvironment with significant focal adhesion development, maturation and also enhanced in vitro cell growth. In conclusion, this work successfully confirmed the feasibility of both strategies for the fabrication of new sol-gel-based hybrid scaffolds with osteoconductive properties.

## 1. Introduction

Three-dimensional (3D) porous scaffolds are widely used in various biomedical ap-plications such as drug delivery [1], cell culture studies [2] and regenerative medicine [3,4,5,6]. Therefore, many different scaffold fabrication methods have been explored to produce biomaterials with the required 3D architecture including not only conventional subtractive approaches such as solvent-casting [7], particulate-leaching [8] and gas-foaming processes [9] but also advanced techniques, such as electrospinning [10] and additive manufacturing (AM) technologies [5,11,12]. Among the various AM procedures, special attention has been paid to the robocasting of porous scaffolds with customized design for bone tissue engineering [13,14,15,16,17]. In general, their architectures mimicking the extracellular matrix (ECM) of trabecular bone are expected to perform better as bone artificial implants because their composition can be adjusted by selecting appropriate ink precursors [18]. In this context, functional requirements such as biocompatibility and bioactivity, that are needed to accommodate cells and guide their growth can be facilitated in a variety of ways depending on the preferred precursors [19,20].

First approaches to the robocasting of such 3D structures were devoted to the processing of ceramics, mainly calcium phosphate compounds due to their osteoconductive and bioactive nature [21,22]. Hydroxyapatite (HA) and β-tricalcium phosphate (β-TCP) were used to prepare aqueous pastes for robocasting [13,14,21,22,23,24,25,26]. To improve the stabilization of the ceramic particles in these water-based inks, some dispersant additives such as polyacrylate (PAA), polyvinyl alcohol (PVA) and polyethyleneimine (PEI) were used [27,28]. Similarly, dispersant or plastifying agents, such as ethyl cellulose or carboxymethyl cellulose, have been incorporated to enhance the printability [29]. As a consequence, many of these inks turned out to be unstable, due to pH and chemistry environment variations. In addition, thermal calcination must be conducted after printing to eliminate undesired organics, followed by sintering to obtain the final ceramic products. These post-process heat treatments are usually accompanied by significant shrinkage of the scaffolds.

Gel-embedded suspensions based on physical hydrogels and organogels from natural biopolymers have been widely reported as suitable solvents in formulating robocasting inks due to their surfactant properties and easy handling [15]. Pluronic^®^ F127 (SigmaAldrich, Saint Louis, MO, USA) commercial sol-gel agent and some cellulose-derived compounds, both exhibiting thermal reversible gelation, are among the most suitable hydrogel compounds utilized in the development of dense ceramic structures by robocasting [20,27,29,30]. Additionally, organogels, whose solidification mechanism depends on the sol-gel transition through solvent evaporation just after printing, have been widely studied in the formulation of ceramic pastes for DIW, including alginate [31,32], chitosan [33,34,35] and gelatin [31,36,37]. Similarly, hybrid organic/inorganic materials [19,38] and polymer/ceramic composite scaffolds, such as polylactide acid or polycaprolactone with high HA content [33] and chitosan/bioglass [39], were fabricated using robotic-assisted deposition at room temperature as bone substitutes, providing favorable substrate conditions for osteoconduction.

Additionally, while being less common, an alternative approach based on sol-gel inorganic polymerization, involving irreversible gelation to determine the printing window, was proposed as solvent media to develop new sol-gel inks for DIW. However, a stiff gel was rapidly formed avoiding an effective deposition of the filaments, according to CAD design [40]. Nevertheless, some different 3D oxide and hybrid organic/inorganic structures have been developed by robocasting using sol-gel inks based on organometallic and biopolymer precursors, with critical control of the irreversible gelation process involving chemical polymerization reactions [41,42]. By this way, silica/chitosan/HA hybrids [42] and TiO_2_ 3D scaffolds for in vitro cell growth have been obtained [43].

Although, in a bulk monolithic state, similar types of sol-gel hybrid materials (xerogels and aerogels) are of particular interest because they have previously demonstrated both appropriate mechanical and biological properties for tissue engineering applications without needing to perform further calcination or sintering heat treatments [44,45]. Hence, the hybridization of silica and biopolymer precursors in the sol state (such as chitosan, gelatin, etc.), can lead to robocasting inks undergoing irreversible gelation, whose viscosity and rheological properties can be tuned without intermediation of chemical binders, just by adding ceramic solid reinforcing phases (e.g., calcium phosphate compounds). By this way, 3D organic–inorganic hybrid/ceramic composite scaffolds, which cannot be achieved by either organic polymers or ceramics separately, can be obtained. To our knowledge, the consideration of whether sol-gel systems exhibiting irreversible gelation can be used to promote solidification in robocasting, thus facilitating the stabilization of printed structures, is a subject that has been scarcely investigated, and this work aims to contribute to this research topic by introducing the first results based on silica/gelatin/β-tricalcium phosphate (TCP) 3D scaffolds.

Otherwise, an alternative rapid prototyping production of 3D microstructures that has also been widely used for biomedical applications is surface micromachining by applying intense ultrashort pulsed laser ablation [46]. It is essentially a technique that has been used for selective material removal to generate precise biomimetic patterning on biomaterials [46,47]. Moreover, the creation of surface micro-channels or micro-holes can be achieved without excessive heating or the appearance of cracks in the annular melting zone [48]. Recently, functional surfaces from collagen gel [49], biopolymer/ceramic composite thin films [50] and metals [51] have been modified through femtosecond laser treatment, leading to 3D structures with potential interest in the improvement of cellular adhesion and tissue engineering applications [52]. Hydrogel 3D structures for biomedical applications have been successfully processed via femtosecond laser pulses [53]. However, although laser-assisted fabrication offers high spatial resolution on surface biomaterial patterning and cell guidance, it suffers from low processing speed and limited scaffold size (usually restricted to the millimeter or micrometer scales) [52,54].

In summary, the aim of this work is to introduce two different strategies to easily obtain 3D sol-gel-derived ceramic/biopolymer porous scaffolds. To this end, first, we present a direct ink writing (DIW) procedure to print 3D porous structures from a suspension of β-TCP microsized powder (2 μm) in a silica/gelatin hybrid sol. A crosslinking agent, 3-glycidoxypropyltrimethoxysilane (GPTMS), is incorporated into the hybrid for enhancing the chemical stability and mechanical properties of the resulting hybrid material and for controlling the degradation rate in aqueous media. As reported in previous results, a similar silica/gelatin hybrid system in combination with the GPTMS coupling agent presented the benefit of biocompatibility, as well as chemical and mechanical stability [55,56]. Next, the ink formulation is optimized to become a paste with adequate viscosity and shear-thinning behavior so that it can easily flow through the nozzle to form continuous rod-like filaments under room temperature conditions, while the resulting scaffolds should have the ability to retain their shapes. The solidification of the printed objects is achieved by irreversible gelation through acid-catalyzed condensation and polymerization of the hybrid matrix sol, followed by drying at ambient temperature. Immediately after, the scaffolds are available for different characterization experiments and cell culture test. Alternatively, the second method makes use of an ultrafast laser characterized by ultrahigh peak power pulses, to introduce a spatially patterned macropore system by laser drilling into a silica/gelatin/ β-TCP sol-gel-derived monolith composite. According to the existing literature, although the technique has been previously utilized for the removal of organically modified silicate coatings [57], this is the first time that a complete perforation by laser micromachining of a sol-gel-processed hybrid polymer/ceramic monolith composite, is planned. Both the hole quality and maximum ablated depth are optimized by controlling the laser machining parameters for the selected solid target. Likewise, the mechanical behavior of the scaffolds is studied under uniaxial compression. In addition, this work investigates the textural properties of the sol-gel biomaterial utilized in scaffolding, as well as its biodegradation in saline solution and osteoblastic response in vitro in the presence of biomaterials.

## 2. Results and Discussion

### 2.1. Synthesis of the Sol-Gel Ink and Fabrication of Scaffolds

In our study, hybrid silica/gelatin matrix sols (SG) were prepared by the sol-gel method, using TEOS as the silica source under acidic conditions. Hybridization between the organic and inorganic phases was facilitated by adding GPTMS, an organosilane crosslinking agent. Gelatin, previously functionalized with GPTMS, is covalently linked to silica thanks to the condensation reaction of the carboxylic acid groups of gelatin with the epoxy ring of GPTMS and condensed into the silica network via Si-O-Si covalent bonding to form hybrid mechanically strong materials [55]. Hybrid sols (SGx) with three different gelatin contents (x = 20, 40 and 60 wt%) were thus prepared. Total gelation time is a critical variable for the solidification process in robocasting [53,58,59,60]. In the current study, it decreased with an increase in the gelatin content. Sols containing the highest gelatin content (SG60) gelled completely after ~5 min at room temperature, while the gelation of sols with 20 wt% gelatin (SG20) took over 60 min. Furthermore, a gel time of ~30 min was observed for the SG40 hybrid matrix, which best fits the robocasting process. Subsequently, to provide appropriate rheological behavior for extrusion to the ink precursor solution, different amounts of β-TCP microsized powder were added (40, 50 and 60 wt%), and after several trial experiments, a loading content of 60 wt% was found to be optimal for printing at room temperature.

**Figure 1:**
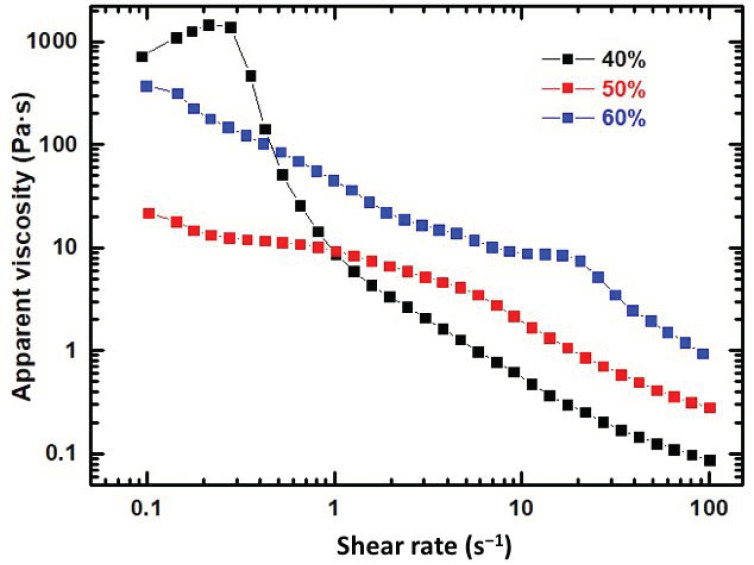
Apparent viscosity of SG40TCPy (y = 40, 50 and 60 wt% β-TCP) pastes vs shear rate.

The rheological properties of the three resulting homogenized pastes were investigated, and Figure 1 shows the apparent viscosity as a function of the shear rate immediately after its preparation. As observed, all of the pastes exhibit a shear-thinning behavior from 0.1 to 100 s^−1^, mainly for higher solid contents. Similarly, the slight increase in the apparent viscosity of the 40 wt% sample at 0.3 s^−1^ shear rate could be attributed to some structural remodeling in the hybrid sol suspension requiring an extra applied stress before it drops due to shear-thinning [30]. This considered, the increase in the viscosity with increasing additions of β-TCP, which can be observed specifically within the 1–100 s^−1^ shear rate range, confirms that, as expected, the sol-gel ink with the highest β-TCP content (60 wt%) was the best option for direct writing.

By this way, 3D scaffolds with dimensions of 10 × 10 × 5 mm^3^ named as SG40TCP60-RC were obtained by the deposition of perpendicular layers of parallel rods with a 610.0 ± 18.0-micrometer diameter in a paraffin bath to reduce non-uniform drying throughout the printing process.

**Figure 2:**
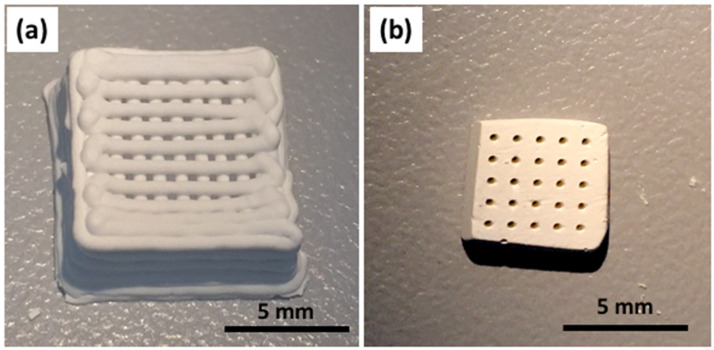
Optical images of 3D macropore structures. (**a**) SG40TCP60-RC scaffold, manufactured by robocasting and (**b**) SG40TCP60-LM, fabricated by laser micromachining (2-millimeter thickness).

Alternatively, a bulk monolith was also produced by solvent casting and evaporative drying using the SG40TCP60 sol-gel paste. After that, it was further subjected to laser micromachining (LM) by ablation in air, in order to determine the minimum thickness to achieve a complete perforation of the sample, aiming to create a regular 350-micrometer diameter macropore system with a rectangular geometry and high accuracy. Some examples of the fabricated RC and LM scaffolds are shown in Figure 2. Both presented the benefit of moderate temperature treatment when using these types of biomaterials for biomedical applications, considering that sterilization treatments involve temperatures lower than 130 °C.

**Figure 3:**
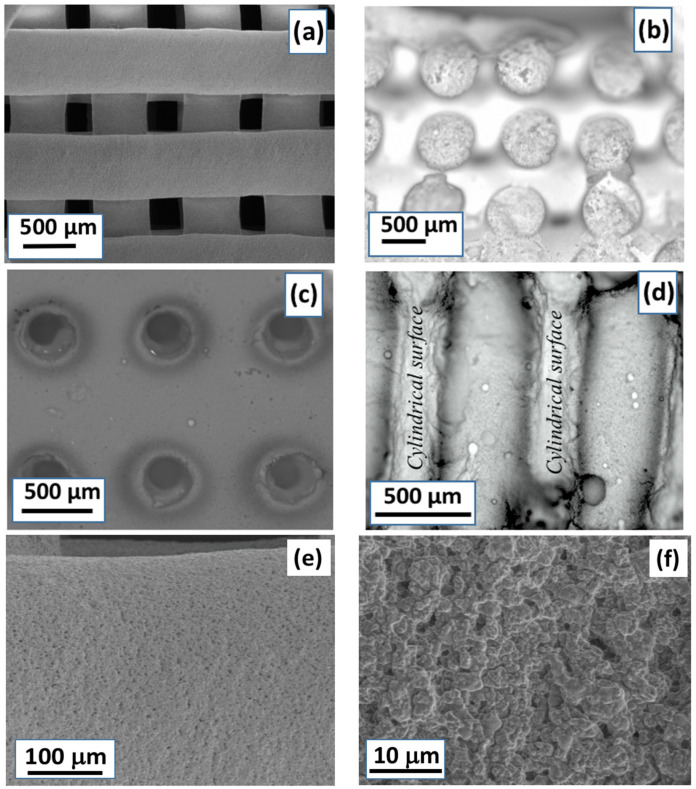
Micrographs show upper scaffold surfaces taken by SEM prepared from: (**a**) robocasting (RC) and (**c**) laser micromachining (LM). Images (**b**,**d**) were made using a confocal microscope and correspond, respectively, to cross-sectional areas of rods obtained by robocasting and axial-section area of the cylindrical pore cavity created by laser ablation (cylindrical surfaces); (**e**,**f**) SEM magnifications of the cylinder rod RC scaffold surface previously shown in Figure 3a.

The SEM micrographs in Figure 3 show the structure and organization of both RC and LM scaffolds at low and high resolution. As observed in Figure 3a,b, the cylindrical cross-sections of the rods remained almost unchanged after extrusion. Therefore, no layer collapsing was observed, and the RC scaffolds maintained their predesigned 3D structure, exhibiting an interconnected macroporosity, according to previous investigations [61,62].

Otherwise, the resulting macropore system introduced by laser machining through a massive SG40TCP60 monolith composite can be seen in Figure 3c,d. Figure 3c shows a cross-sectional view of the cylindrical holes with 350.8 ± 16.6-micrometer diameter oriented parallel to the cylinder axes, whose centers are regularly spaced by 1312.0 ± 23.0 μm, created by laser ablation on a 1 cm^2^ flat surface of a 2 mm thick rectangular composite specimen. Moreover, Figure 3d shows the axial-section of the cylinder holes, passing through and maintaining its diameter along the entire thickness. This procedure is preliminary research of a deeper investigation in which the progression of the porosity through thicker specimens as well as its interconnection is under development.

Figure 3e,f provide visual clues about the surface texture of the solid phase. It appears to consist of agglomerates of ceramic microparticles, embedded by a thin film of silica–gelatin hybrid polymer wetting the contact surfaces between ceramic particles, presumably filling and sealing the interparticle pore walls and, therefore, provoking pore blocking effects in the characteristic micro-mesoporosity of this type of gel. This structure agrees with the physisorption results.

### 2.2. Physical and Textural Characterization

RC and LM 3D scaffolds were fabricated displaying negligible volume shrinkage (see Figure 2) using the same hybrid sol-gel paste composition. Therefore, the macroporous interconnected structure of the RC scaffold fabricated by the deposition of perpendicular layers of parallel rods was preserved with minimal deviations from its respective CAD design. The same observation applies for the LM scaffold under laser processing, for which a system of parallel cylindrical pores by the ablation of material was produced. A highly uniform surface microstructure of both RC and LM scaffolds was observed by SEM (see Figure 3), confirming the homogeneity of the scaffold skeleton at the microstructural level.

The apparent densities for the RC and LM scaffolds were calculated from geometric measurements and similar results were observed: 1.00 ± 0.01 g cm^−3^ for RC scaffolds and 1.11 ± 0.01 g cm^−3^ for LM scaffolds. Otherwise, the skeletal density measured by He pycnometry for an SG40TCP60 cylinder monolith was 1.97 ± 0.31 g cm^−3^, which underestimates by 20% the value determined by the rule of mixtures, bearing in mind the composition of the resulting material: 40 wt% [SiO_2_ Gelatin (40 wt%)] and 60 wt% β-TCP. This result revealed the existence of a closed non-connected porosity within the material in the micro-mesopore range. As a consequence, the total open porosity of the samples calculated from Equation (1) was 50 ± 5% for the RC scaffolds and 44 ± 4% for the LM scaffolds.

**Figure 4:**
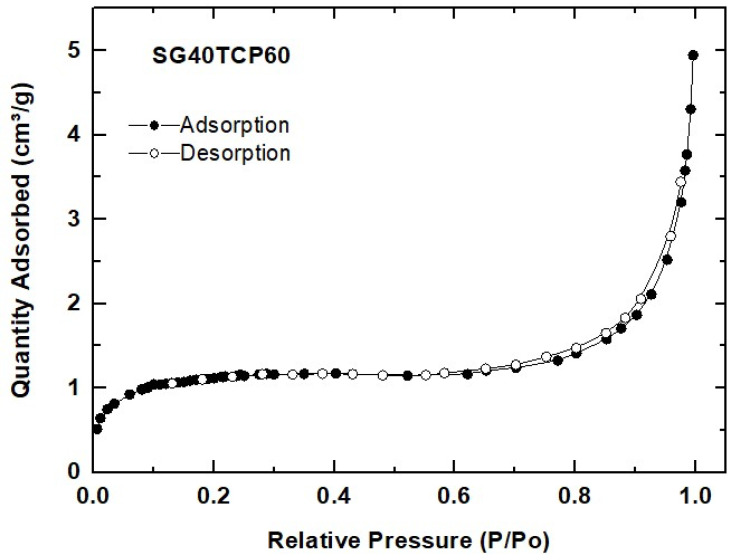
N_2_ physisorption isotherm measured for the SG40TCP60 material.

N_2_ physisorption experiments revealed some microstructural features of the SG40TCP60 material used for scaffolding. According to IUPAC classification, the sample exhibited reversible Type II isotherm, characteristic of macroporous or non-porous solids, as observed in Figure 4. The corresponding curve shape is attributed to unrestricted monolayer–multilayer adsorption up to high p/p_0_, while the thickness of the adsorbed multilayer normally develops an apparent increase without limit when p/p_0_ = 1 [63]. This type II N_2_ isotherm usually displays a similar form over a wide range of multilayer coverage. However, differences arising in the curvature can be attributed to a significant overlap of monolayer coverage and multilayer adsorption, caused by the simultaneous presence of both high- and low-energy surface sites [64]. As a result, the N_2_ adsorption process in SG40TCP60 can be described in terms of the BET parameter C data collected in Table 1. Consequently, higher values of C (~80–120) reveal that the adsorption process on energetic surfaces is predominant and the nitrogen monolayer is completed at low p/p_0_ [63]. By contrast, low-energy adsorbents exhibit lower C values reflecting an appreciable monolayer–multilayer overlap [64]. These situations apply in the case of SG40TCP60, with a C value becoming even negative and, therefore, meaningless, thus invalidating the application of the BET method due to the difficulty of knowing the specific monolayer capacity. Special attention has been given by other authors to the preparation of new gels and composites in the silica–gelatin system, and the mutual interactions of organic and inorganic components on the formation of the hybrid network have almost been completely elucidated [65]. Research efforts have been primarily focused on the preparation of highly silica–gelatin porous materials, including crosslinkers such as GPTMS, mainly by controlling the sol-gel drying post-processing steps (supercritical drying) [66,67] or by thorough extraction of gelatin with water from xerogels [68] without using any crosslinker. In all these cases, specific surfaces in the range of 400–700 m^2^g^−1^ were obtained for gelatin content up to 30 wt%. However, by varying the pH and processing conditions, xerogels with surface areas as low as 4–6 m^2^g^−1^ were obtained, consisting of silica nanoparticles embedded in a gelatin network [68]. The textural properties measured for the SG40TCP60 material point in this direction, which means that both ceramic β-TCP microsized and silica nanosized particles are embedded in a gelatin percolating network.

Hence, as a general conclusion, it can be stated that regardless of its content, gelatin drastically alters the microstructure of the SG40TCP60 hybrid sample during drying in air by sealing the micro-mesopore distribution, thus provoking a severe reduction in the pore volume and causing the almost total loss of the specific surface area. The results and descriptions are consistent with the above-mentioned skeletal density measurements carried out by He pycnometry.

### 2.3. Mechanical Characterization

**Figure 5:**
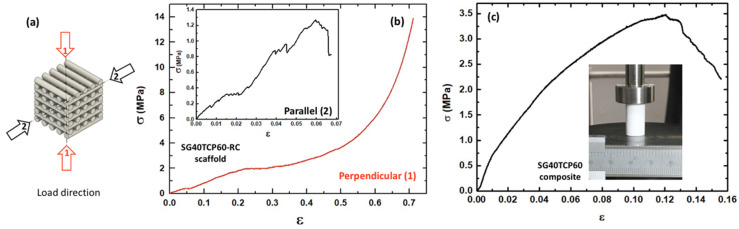
Stress–strain curves from the uniaxial compression test: (**a**) load directions; (**b**) SG40TCP60-RC scaffold in perpendicular and parallel (inset) load directions to the printing plane; (**c**) SG40TCP60 composite cylinder specimen.

Compressive strength, compressive modulus of elasticity and other aspects of the stress–compression relationship were investigated by the uniaxial compressive stress on rectangular prisms obtained by robocasting (SG40TCP60-RC). To evaluate the directional mechanical strength and to determine the effect of structural compaction on its mechanical anisotropy, the samples were loaded in two different directions (perpendicular and parallel to the printing plane), as described in Figure 5a. Figure 5b shows the representative nominal stress–strain curve from the uniaxial compressive test performed on the perpendicular loading direction. It consisted of a short linear stage (0–22% strain), followed by load stabilization (2.0 ± 0.3 MPa) until 30% strain, corresponding to the collapse of the printed layers. Then, as the structure gets more compacted causing the characteristic pile-up of layers, the stress–strain curve increases progressively even after very large strains (~70%) until the whole structure is totally destroyed, and maximum load does not correspond to the compressive strength. This relationship between the stress and strain is related to the 3D structure of the scaffold, revealing differences in the mechanism of fracture and properties reported for similar samples [61], underlining the relevance of this new fabrication procedure. The inset in Figure 5b shows a representative stress–strain curve registered in the parallel direction, presenting heterogeneous transfer and distribution of the applied load, attributed to layer debonding due to a weak structural compaction of the sample. An abrupt brittle failure behavior at about 1.2 ± 0.3 MPa and 6.7% strain reports the existence of some mechanical anisotropy between the two loading directions.

In order to determine the mechanical behavior of the bulk samples being subjected to laser ablation (SG40TCP60-LM), additional compressive tests were also performed on the SG40TCP60 composite cylinder specimens. A representative stress–strain curve is shown in Figure 5c for test specimens of cylindrical geometry (10-millimeter diameter, 20-millimeter height), thus implementing the ASTM D7012 standard. Typical elasto-plastic behavior of sol-gel hybrid matrices reinforced with a high content of solid microparticles is displayed, where the relatively low compressive strength is provoked by a weak interfacial interaction between the matrix and the filler. A maximum stress of 3.30 ± 0.3 MPa was observed at 12% strain while Young’s moduli obtained at the beginning of the curve showed marked stiffening in comparison with the printed sample. Thus, Young’s moduli of 12.7 ± 5.2 MPa and 18.0 ± 2.7 MPa were estimated for perpendicular and parallel loading directions of the printed scaffold, respectively, while 56.3 ± 14.5 MPa was observed for the cylinder monolith sample. Young’s modulus, as well as compressive strength and maximum compressive strength results of the different mechanical configurations, is reported in Table 2.

The scaffold obtained by laser micromachining (2-millimeter thickness) was not tested in compression because, according to the ASTM D7012 norm, the specimen must have a diameter of 1 mm to be tested. Hence, the related circular area does not allow for the creation of a hole size distribution system large enough to be representative of the laser-ablated scaffold design (350-micrometer diameter hole, whose centers must be spaced by 1015 μm). Nevertheless, further investigation of the laser processing to progress through thicker specimens would facilitate the measurement of the compressive strength.

### 2.4. Degradation Behavior in PBS

**Figure 6:**
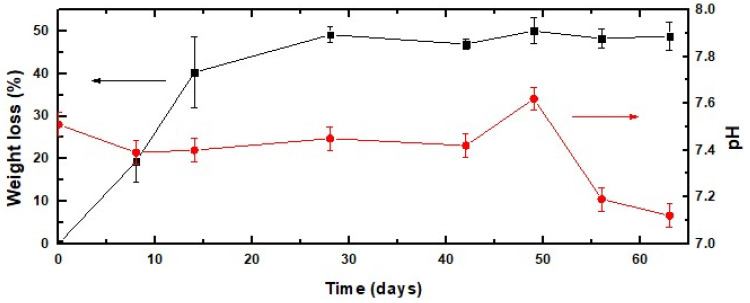
Weight loss (-■-) of the SG40TCP60 material in PBS and pH evolution (-■-) of the solution for 9 weeks.

As bone regeneration is facilitated by the release of calcium and phosphorus ions that regulates the activation of osteoblasts and osteoclasts, the study of the biodegradation rates of these biomaterials is of crucial importance in future clinical applications. Figure 6 shows the dependence of the weight loss of the SG40TCP60 material on the incubation time for over 9 weeks. It occurs in two stages: a relatively fast first step during the first two weeks of soaking in PBS, where the scaffold exhibited an approximately 40 wt% weight loss and a slower second 10 wt% weight loss that took place during the next two weeks. Following this, the sample weight remained almost constant over the remaining test period until reaching the end of the test, in agreement with the results reported for similar hybrid gelatin-based materials [55,69]. This considered, the pH values were monitored over the same experimental time point as the weight losses, and they are also shown in Figure 6. As revealed, only small variations in the pH value were observed after the degradation process, varying from 7.54 at the starting point of the experiment to 7.18 at the end (week 9) of the test. 

**Figure 7:**
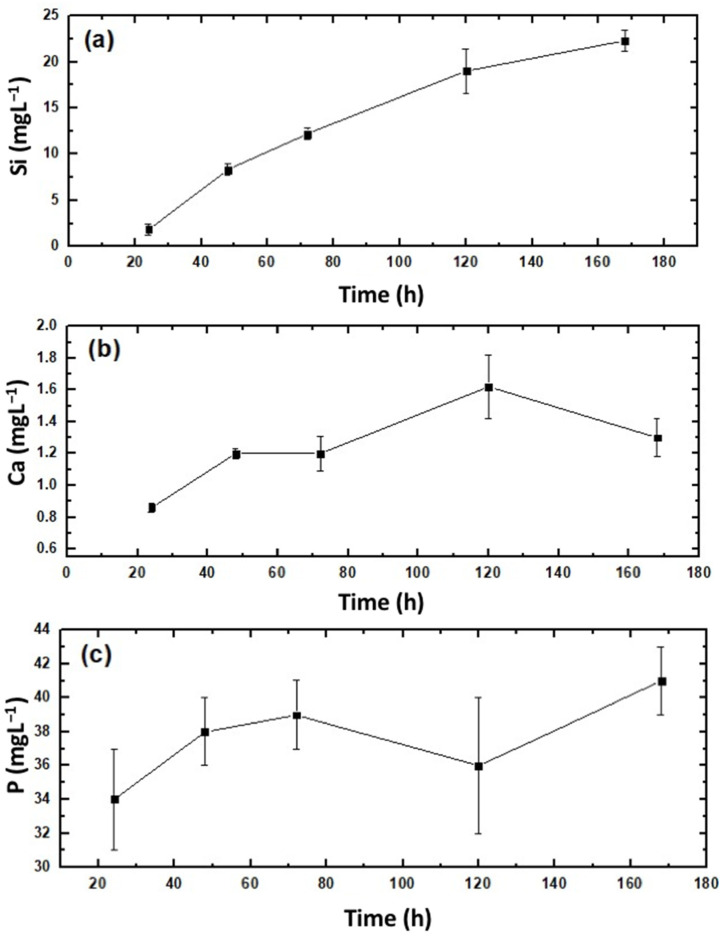
Profiles of cumulative ion release of (**a**) Si; (**b**) Ca; (**c**) P from the hydrolytic degradation of the SG40TCP60 material after immersion in PBS for 1 week. Both free Ca- and P-plotted concentrations were obtained by subtracting the initial Ca and P concentrations of PBS from the ICP_MS measurements after different immersion periods.

The release profiles of Si, Ca and P from the 3D-printed scaffold soaked into the PBS buffer solution were evaluated using ICP_MS, and their corresponding results are presented in Figure 7a–c, respectively. A continuous increase in the Si concentration in time in the buffer medium was observed, from zero to approximately 23 mg L^−1^, during 7 days of soaking (Figure 7a). This was attributed to a slow hydrolytic erosion process of the material in contact with the environment. These things considered, the quantified Si ion concentration release was three times lower than the Si concentration observed for other mesoporous silica-based hybrid xerogels made without gelatin using a similar experimental procedure [70]. The results obtained for both Ca and P release were obtained by subtracting the initial Ca and P concentrations of PBS [71] [3.23 ± 0.30 mg/L (Ca) and 273.00 ± 6.00 mg/L (P)] from the ICP_MS measurements in the studied mediums after different immersion periods. The amount of Ca released is presented in Figure 7b, exhibiting a slight increase from above 0.8 mg L^−1^, after 24 h of exposure to PBS, up to 1.3 mg·L^−1^ at the end of the 7th week. Figure 7c shows the release profile of P, revealing a high dissolution from 0 to above 34 mg L^−1^ during the first 24 h, then increasing during the next 24 h up to 38 mg L^−1^ and remaining almost constant until reaching above 40 mg L^−1^ at the conclusion of the test procedure.

Basically, the Si-released ion concentration found in PBS was in good agreement with previous reports from silica–gelatin-similar hybrid materials [55], a result that potentially enhances osteogenesis [72]. Otherwise, Ca- and P-released concentrations were found in a typical range regulated by the small solubility of β-TCP (0.25 mg/L at 25 °C) [73], known well as a resorbable bone repair [6].

### 2.5. Cell Culture

Culture cells were tested on the SG40TCP60 composite cylinder biomaterial in order to assess the effect on cell viability, cell–biomaterial interactions and osteoblasts growth and maturation of the base compound used in scaffold manufacturing. Cell viability at seeding was up to 98%, and no significant apoptotic phenomena were detected either in the control or experimental groups. The scaffolds were designed to allow and improve cellular growth and ideally to favor positive interactions between the cells and materials, leading to the formation of functional tissue for medical purposes [6,74].

**Figure 8:**
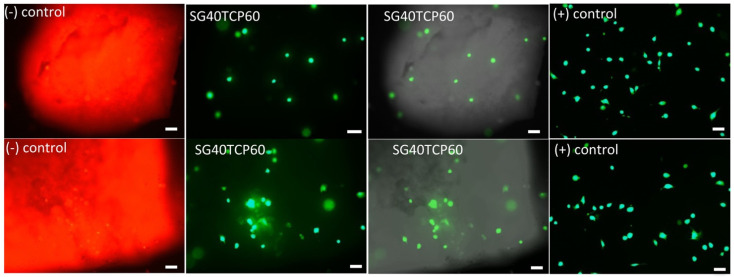
Live/Dead staining of HOB^®^ cells after 48 h in culture. Positive controls for control groups shown in last column. Images acquired in the fluorescence and Nomarski modes of a Leica DMLI LED inverted microscope. Live cells (green); dead cells (red); biomaterials (gray). Scale bar equals 20 μm.

**Figure 9:**
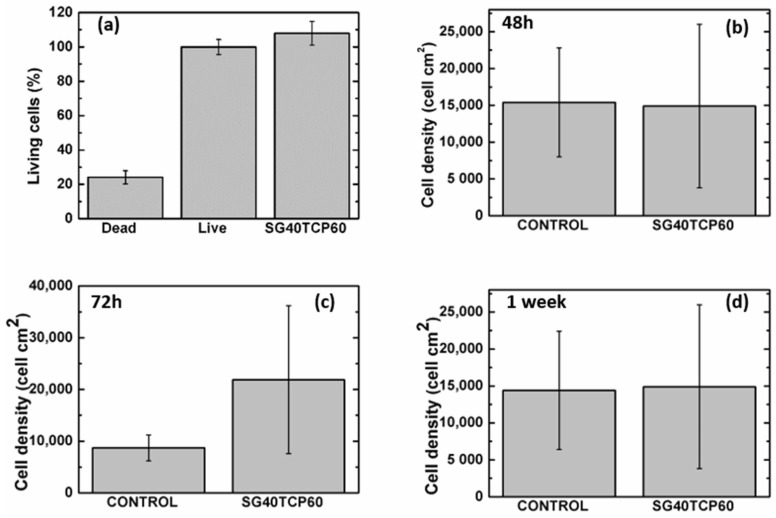
(**a**) Quantitative data for live dead assay after 48 h in culture and cell density for experimental times of (**b**) 48 h, (**c**) 72 h and (**d**) 1 week. One-way analysis of variance. Statistical significance *p* < 0.05.

Cell live dead viability assays (live dead^®^) revealed higher percentages of viable (green) than dead (red) cells after 48 h (see Figure 8). In the osteoblast population, a majority of cells migrate towards and then adhere to biomaterial, crowding on top while a few osteoblasts remained adhered to the well bottom. No significant differences with positive control were observed either in terms of the cell viability or cell density (see Figure 9).

### 2.6. Cell Morphology, Cytoskeletal Organization and Focal Adhesion Distribution

**Figure 10:**
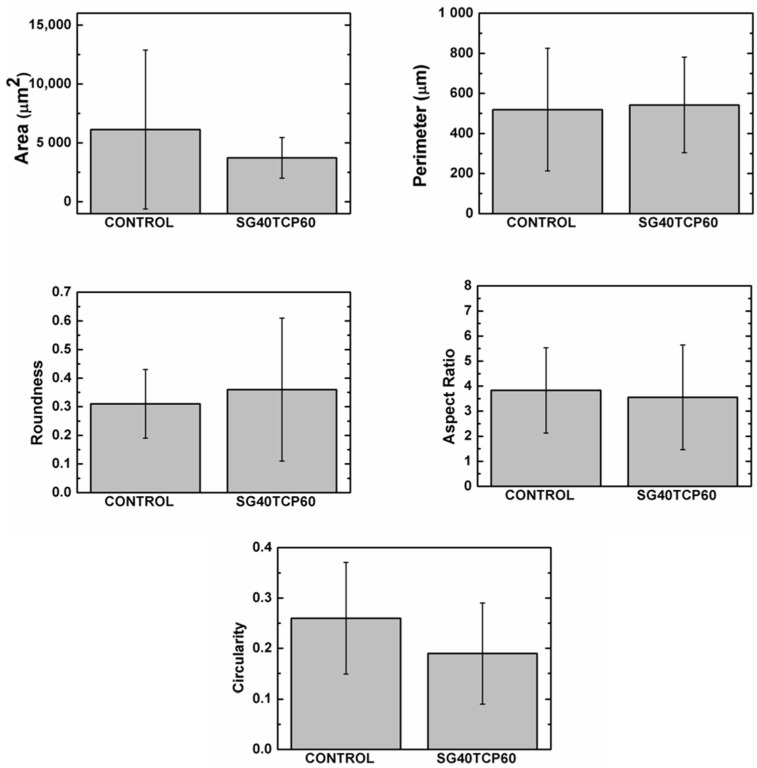
Quantitative data for shape parameters after 48 h. One-way analysis of variance. Statistical significance *p* < 0.05.

The ideal scaffold properties should allow for an adequate microenvironment for cell attachment, proliferation and differentiation, but also provide an adequate pathway for the nutrients to approach the cells. Furthermore, the scaffold properties should guarantee an optimal mechanical support for the cells, together with an adequate control of the degradation rate and absence of cytotoxicity. As described, live dead assays revealed a careful cell distribution on top and along the SG40TCP60 material pieces (see Figure 8). It was confirmed that osteoblasts seeded on the scaffolds presented an initial polarization from the first 24 h, with subsequent cell adhesion and changes in cell morphology compatible with osteoblast differentiation [44,74,75,76,77,78,79,80]. This was confirmed by changes in shape parameters, mainly those observed in area, roundness and circularity quantifications (Figure 10).

**Figure 11:**
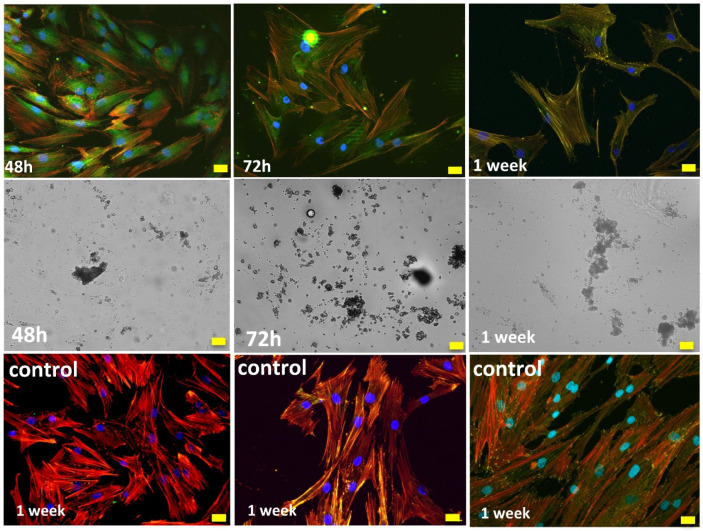
HOB^®^ Osteoblasts growing in the presence of the SG40TCP60 biomaterial. Images acquired in the confocal microscope in the fluorescence (upper row) and Nomarski mode (mid row). In red: actin cytoskeleton immunolabeled with rhodamine phalloidin, in green: vinculin immunolabeling for focal adhesions and in blue: DAPI-labeled nuclei. Representative images of control groups growing on glass are shown in the lower row. Scale bar equals 20 μm.

Immunolabeling for actin cytoskeleton after 48 h revealed that in comparison to control cells, osteoblasts elongated in the presence of SG40TCP60, while actin cytoskeleton developed and small focal adhesions became distributed along the cell (Figure 11). After 72 h in culture and in the presence of SG40TCP60, actin cytoskeleton mainly develops in the periphery with small focal adhesions and filopodial and lamellipodial emissions, while some cells elongate and some others grow wider. A remarkable number of stress fibers arise into a well-developed actin cytoskeleton mostly enhanced in the periphery with focal adhesions widely distributed, predominantly small-sized and located on the tips of stress fibers. A progressive approach of osteoblasts to the biomaterial was observed with time, and the material surface appeared to be covered, while cells contacted to neighbors and to the material surface. No stress fibers or polarization were found in the control cells. After 1 week in the culture, filopodial emissions were predominant and mature focal adhesions on the tips of widely distributed and well-developed stress fibers became evident. The phase contrast mode reveals that osteoblasts, independent of number or size, delicately distributed on the biomaterial at any experimental time.

## 3. Conclusions

A novel biocompatible 3D scaffold was successfully fabricated by robocasting using a sol-gel paste ink, consisting of 60 wt% β-TCP powder dispersed in a silica/40 wt% gelatin hybrid matrix (SG40TCP60), undergoing an irreversible gelling process. The printing procedure was performed without needing to incorporate chemical additives, which simplifies, to a great extent, the overall printing process. Moreover, the technique is adaptable enough to allow ink formulation from a broad range of materials, just by adjusting its gelation time and selecting an appropriate rheology of the resulting sol-gel paste because the processing is performed at room temperature. SG40TCP60 3D scaffolds with accurately controlled pore size (about 350 μm) with interconnected porous structures and negligible volume shrinkage were thus obtained. In addition, a novel 3D scaffold consisting of parallel regularly spaced cylindrical holes of about 350-micrometer diameter created on 2 mm thick SG40TCP60 bulk monoliths was successfully fabricated by laser micromachining. The method is being researched as an alternative manufacturing method for 3D structures from a variety of sol-gel materials. The compressive behavior of the laser-assisted scaffold was found to be in the range of the trabecular bone of similar skeletal density, with significant mechanical anisotropy in the case of the robocasted scaffold. Moreover, the SG40TCP60 biomaterial underwent in vitro biodegradation, and the release of Si, Ca and P ions to the free medium while its osteoblast response demonstrated that it is composed of a biocompatible material. In addition, and for clinical use, these scaffolds are easily sterilizable and their 3D architecture facilitates cell attachment, proliferation and initial markers of differentiation such as morphological changes and stress fiber and focal adhesion development. Further studies are needed to discern how the surface topography could influence osteoblast response in the culture for personalized scaffold design.

## 4. Materials and Methods

### 4.1. Materials

Tetraethylortosilicate (TEOS, 99%) and hydrochloric acid (HCl) (37%) were purchased from Alfa Aesar (Haverhill, MA, USA). Gelatin (from porcine skin, Type A, gel strength 300) and 3-glycidoxypropyltrimethoxysilane (GPTMS, >98%) were both purchased from Sigma Aldrich (St. Louis, MI, USA). Microsized β-tricalcium phosphate (Captal β-TCP, 2.0 μm average particle size) was purchased from Plasma Biotal Ltd. (Buxton, Derbyshire, UK). Ethanol absolute (99.5%) was purchased from Panreac (Spain). HOB^®^ human osteoblasts, foetal calf serum and Osteoblast Growing Medium were purchased from Promocell (Heidelberg, Germany). PBS, methanol, Triton x-100, bovine serum albumin, paraformaldehyde, rhodamine phalloidin and monoclonal anti-vinculin FITC conjugate were all acquired from Sigma, (St Louis, MI, USA) and Hard Set Vectashield with DAPI ^®^ from Vector (Burlingame, CA, USA).

### 4.2. Scaffold Fabrication

**Figure 12:**
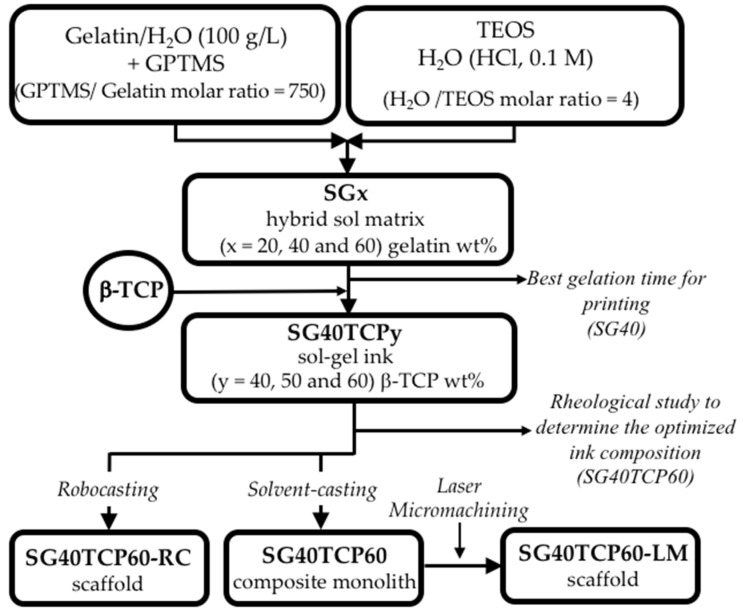
Schematic showing the different processing methods used to prepare SG40TCP60 3D sol-gel-derived scaffolds by robocasting (RC) and laser micromachining (LM). The last-mentioned scaffold was fabricated by laser irradiation of the corresponding monolith composites, which in turn were obtained by solvent casting of SG40TCP60 ink, followed by evaporative drying, (y = 60 wt% represents the β-TCP content for the optimized ink composition according to rheological studies.)

In Figure 12, a flow chart summarises the process used in the scaffolds fabrication. 

#### 4.2.1. Preparation and Characterization of the Silica/Gelatin/β-TCP Sol-Gel Ink for Robocasting

A gelatin solution in 80 °C distilled water (100 mg mL^−1^) was prepared under vigorous agitation for 10 min. After cooling at room temperature, functionalization of gelatin was promoted by reaction with GPTMS to give a molar ratio of 750 with respect to gelatin. The silica sol was obtained separately by mixing TEOS with a stoichiometric quantity of acidified water (HCl, 0.1 N) to obtain a transparent silica sol. Next, both gelatin-GPTMS and TEOS solutions were mixed and homogenized to produce silica/gelatin hybrid sol matrices, labeled as SGx, with x = 20, 40 and 60 wt% gelatin content. All chemical reactions were carried out at room conditions with mechanical stirring. The gelation time of SGx gels decreased from 60 to ~5 min with increasing gelatin content from 20 to 60 wt%, respectively. Therefore, given that the setting time and rheological properties are critical for the direct writing process, the SG40 hybrid was considered to exhibit the best value of gelation time desirable for successful printing (~30 min), while SG20 and SG60 with, respectively, longer or shorter gelation times were not used in the ink formulation. Next, three different microsized β-TCP high powder contents (40, 50, 60 wt%) were added to the selected SG40 hybrid sol, until three different solid suspensions were obtained, named as SG40TCP40, SG40TCP50 and SG40TCP60, which were placed in a planetary centrifugal mixer (ARE-250, Thinky Corp., Tokyo, Japan) for 5 min to obtain stable homogeneous solid suspensions. Next, the rheological characterization of the suspensions was performed at room temperature, and the best composition for the ink formulation was selected. The apparent viscosities of the three hybrid pastes prior to aging and gelation were measured with the Discovery HR10 rheometer (TA instruments, USA), using a cone-plate geometry and a gap of 50 µm, for shear rates ranging from 1 to 100 s^−1^ at 25 °C. All measurements were conducted in triplicate.

#### 4.2.2. Fabrication of Silica/Gelatin/β-TCP Hybrid Scaffolds by Robocasting

**Figure 13:**
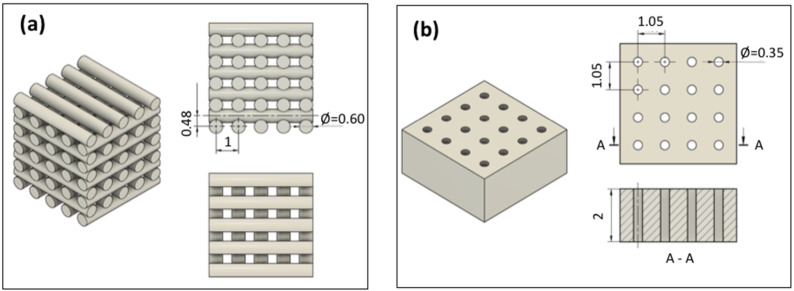
Design section views of the scaffold shapes and its corresponding pore structure: (**a**) layer-by-layer of the interconnected macroporous pattern planned by using robocasting; (**b**) arrangement of the cylindrical holes for a macroporous structure generated by applying laser ablation in a rectangular prism monolith composite. All dimensions are presented in mm.

The optimized ink (SG40/β-TCP 60 wt%, as detailed in Section 2.1) was introduced into the printing syringe and then placed on the three-axis motion stage of an A3200 robotic deposition device (Aerotech/3D inks, Stillwater, OK, USA), which was controlled by a computer-aided direct-write program (Robocad 3.0, 3-D Inks, Stillwater, OK). Deposition of the ink was carried out by extrusion of filament rods layer-by-layer at room temperature with a speed of 20 mm s^−1^, using a conical nozzle with a tip diameter of 600 μm. The scaffolds were created from a CAD model using a control software (RoboCAD 4.1, 3D inks LLC, OK, USA) and consisted of a 10 × 10 mm^2^ squared base, where the distance between filaments was 400 μm. The model comprises 5 layers, always rotated 90° from the previous one, with a distance between layers of 480 μm. Deposition was performed in a paraffin oil bath to prevent non-uniform drying during assembly, while the hybrid paste underwent chemical polymerization to produce solid composite filament rods. This way, a 3D scaffold was obtained, as described in Figure 13a. The resulting sample, labeled as SG40TCP60-RC, was dried in air at room temperature for 24 h and then at 100 °C for 48 h to evaporate organics. Given that the viscosity of the ink increased in time due to the sol-gel condensation and that gelation is the mechanism associated with the shape retention and solidification of the 3D-deposited structures, a 30-minute printing window interval, as found for the SG40 hybrid matrix, was considered long enough to perform the robocasting procedure.

#### 4.2.3. Fabrication of Silica/Gelatin/β-TCP Hybrid Scaffolds by Laser Micromachining

The sol-gel slurry was allowed to polymerize at room temperature in hermetically closed plastic containers, where gelation took place in about 30 min. The resultant gel was aged for 1 week and then dried by evaporation at 50 °C for another 48 h to obtain the related SG40TCP60 monolith composite to be used as the solid target for laser irradiation. Next, laser micromachining of the composite surface was conducted in the lasing processing laboratory of the Condensed Matter Physics Department of the University of Cadiz by using a commercial high repetition rate pulsed laser model Carbide CB3-40W of light conversion (nominal pulse energy beam (Ep) of 38 μJ, changeable pulse width (τp) from 190 fs to 10 ps, an adjustable repetition rate from one single pulse to 1 MHz) equipped with a Carbide harmonics module to generate ultrashort laser pulses with variable wavelength in the range between 1035 nm and 343 nm. This laser system was equipped with galvanometric mirrors, which allowed the movement of the laser radiation along the surface of the target; a mechanical Z-axis that allowed the position of the laser beam focus to be modified with respect to the surface of the sample. This laser device was controlled with a CAD-like LS-PRO software, which allows a precise control of the laser working parameters. In these laser micromachining, the laser beam was incident perpendicular to the longitudinal section of a 10.0 mm × 10.0 mm × 2.0 mm composite rectangular prism in order to generate a pattern of cylinders 350 µm in diameter, whose centers were separated by 1050 µm. To perform this, the laser working parameters were established at: 343 nm (UV) for the pulse laser wavelength, 38 µJ for the average laser pulse energy (0.76 W of beam power), 20 kHz for the pulse repetition rate, 220 fs for the pulse width and 10 mm∙s^−1^ for the scanning speed. In order to drill through the whole thickness of the rectangular prism, the pattern was repeated 3 times in the same spot, and then, the Z-axis was lowered 150 μm. A geometrical model of the perforated monoliths, labeled as SG40TCP60-LM, is shown in Figure 13b.

### 4.3. Characterization Techniques

Physical, textural and mechanical properties as well as biodegradation and biological responses were investigated for the SG40TCP60 composition optimized for extrusion.

The bulk density (*ρ_Bulk_*) was obtained by measuring the dimensions with a sliding caliper and the mass with a microbalance (precision of ±0.1 mg). In addition, the skeleton density (*ρ_Skel_*) of the SG40TCP60 monolith composite was measured using helium pycnometry, according to method described by Weinberger et al. [81], so that the porosity of the scaffolds (ε) could be estimated from Equation (1). Values were obtained from the average of three replicates.

(1) $$\varepsilon =\left(1-\frac{\rho_{Bulk}}{\rho_{Skel}}\right)\times 100$$

Specific surface area, pore volume and pore size were investigated using nitrogen physisorption experiments, considering BET and BJH standard models for the analysis. A Micromeritics ASAP2010 working at 77 K and equipped with a pressure transducer with resolution of 10^−4^ mm Hg was used for the analysis.

The mechanical behavior was characterized through uniaxial compression tests in samples obtained by robocasting (SG40TCP60-RC) using as-printed right-angled prisms with 10 × 10 × 5 mm^3^ dimensions as test specimens, according to ASTM D7012 standard [82]. Compressive loads were applied in the direction perpendicular and parallel to the printing plane of the RC samples. The compressive strength and maximum strain were computed from the maximum stress and deformation before the fracture of the sample, and the Young’s modulus was obtained from the slope at the beginning of the stress–strain curve. For comparison, the compressive behavior of the SG40TCP60 cylinder-shaped monoliths (18.00-millimeter height and 10-millimeter diameter) was also examined. All the tests were conducted in air with a Shimadzu AG-I Autograph 5 kN using a load cell of 500 N within a ±1% of the indicated test force at 1/250 load cell rating and a constant crosshead speed of 0.1 mm min^−1^. For each testing condition, a minimum of 3 samples were examined.

### 4.4. Biodegradation

The in vitro biodegradation study of the SG40TCP60 material was performed in phosphate buffer saline (PBS; pH 7.4), an effective physiological buffer with pH, osmolarity and ion concentrations similar to human plasma [83,84,85]. The weight loss of the samples was determined gravimetrically at room temperature, using a microbalance (precision of ±0.1 mg). The pH time-dependence of the PBS-incubated solution was also monitored using a pH meter probe (HACH sensIONTM + pH = 3, with pH resolution of 0.01). Basically, printed samples were cut with a sterile scalpel knife and then immersed in PBS (10 mg mL^−1^) under physiological conditions at 37 °C. In the next stage, samples were removed from buffer media weekly and then weighed after wiping off any liquid on the surface. Likewise, the pH of the incubated solution was recorded. Last, samples were immersed again to continue with the degradation test in a totally refreshed PBS solution. This procedure was repeated over 9 weeks of incubation period, and the weight loss time-dependence was calculated according to Equation (2), as follows:

(2) $$Weight\;loss\;\left(\%\right)=\frac{\left(W_{i}-W_{t}\right)}{W_{i}}\times 100$$

where *W_i_* and *W_t_* are the weights of the samples before and after the degradation experiment, respectively, determined at regular intervals of 1 week.

In addition, to provide a more detailed picture of the biodegradation process, the concentrations of accumulated of Si, Ca and P ions in the incubated PBS solution were also measured. The corresponding ion release profile analysis was performed by inductively coupled plasma mass spectrometry with an ICP-MS mass spectrometer, Series X2, Thermo Elemental. The ion concentrations were quantified by collecting 4-milliliter aliquots of the scaffold/PBS-incubated solution (1 mg mL^−1^), at intervals of 12 h, 24 h, 48 h, 72 h, 120 h and 168 h after starting the experiment and without refreshing solution. The aliquot parts were filtered with a 0.45-millimeter Millipore membrane filter, subsequently placed in plastic vials to prevent any type of contamination and stored at 4 °C until the ICP-MS analysis. Weight loss, pH and Si, Ca and P ion-released concentrations were calculated using the average of all triplicates of the samples.

### 4.5. Cell Culture

HOB^®^ cells were seeded, under sterile conditions, on the preselected scaffolds. Osteoblasts were detached when the optimal confluence was reached, then counted to optimal cell density and studied for cell viability in an automated Luna^®^ cell counter, Invitrogen. All experiments were performed with cells under ten population doublings. In order to achieve optimal sterilization of the biomaterials prior to cell seeding, a clinically standardized autoclave (under European standard DIN EN ISO 13060 recommendations for class B autoclaves) was employed. Sterilized samples were placed on Mattek^®^ glass bottom wells in a laminar flow chamber. A drop of 50 μL of cell suspension containing 15,000 HOB^®^ cells/cm^2^ was then added to each sample and then kept for 30 min under incubation at 37 °C and 5%. Afterwards, wells were filled with supplemented OGM^®^ (final concentration of 0.1 mL/mL of fetal calf serum). Experiments were performed at 37 °C and 5% CO_2_. The growth medium was changed every two days. Experimental groups included: SG40TCP60 sample and HOB^®^ cells grown on glass under the conditions before mentioned, used as the control.

### 4.6. Live/Dead Cell Assay

In order to evaluate the viability/cytotoxicity of HOB cells grown on SG40TCP60 sample and also in the controls, live /dead cell assay was performed as follows: after being incubated for 48 h, the cell/scaffold constructs were twice PBS-rinsed and then exposed to calcein-AM (0.5 μL/mL) in PBS to display the live cells and then to ethidium homodimer-1 (EthD-1) (2 μL/mL) in PBS to display dead cells, correspondingly. The samples were then observed in the fluorescence and Nomarski modes of a Leica DMLI LED inverted microscope.

### 4.7. Cell Morphology and Spreading

Cell morphology, alignment, distribution and spreading of osteoblasts were daily assessed after examination with the phase-contrast microscope and at the end of any experimental period. Furthermore, the Nomarski mode of both fluorescence and confocal microscopes was combined with the fluorescence mode to assess the simultaneous imaging of both the material and growing cells.

### 4.8. Actin Cytoskeletal Organization

Rhodamine-phalloidin and vinculin immunolabeling was performed after incubation for 48 h, 72 h and 7 days in order to assess cytoskeletal changes and focal adhesion development. Briefly, cells were washed with prewarmed PBS, keeping pH to 7.4 and fixing the constructs with paraformaldehyde (3.7%) at room temperature, and then permeabilized with 0.1% Triton x-100. After twice washing, preincubation with 1% bovine serum albumin in PBS for 20 min was performed, prior to cell immunolabeling with rhodamine phalloidin for 20 min. Then, samples were rinsed twice with prewarmed PBS prior to Vectashield ^®^ mounting. At least five samples were seeded and analyzed per experiment and experimental group: SG40TCP60 sample and control. HOB^®^ cells grown on glass under the conditions above-mentioned were used in the last case.

### 4.9. Confocal Examination

An Olympus confocal microscope was employed to assess the surface influence on the following parameters: cell density, cytoskeletal features and organization, focal adhesion distribution and changes in cell morphology. A total of 50 cells per sample were analyzed at least in each group, for a time interval not higher than 5 min to avoid photobleaching using a pinhole of 1 Airy unit.

### 4.10. Image Analysis

Sample images were collected as frames obtained at 40x magnification at a resolution of 1024 × 1024 Image J software (NIH, http://rsb.info.nih.gov/ij, accesed on 9 October 2020) was used for image processing. Shape variables analyzed were: area, roundness, circularity, perimeter and aspect ratio. A minimum of 40 regions of interest (ROIs) were measured under the following criteria: clear identification of nucleus, well-defined limits and absence of intersection with neighboring cells. All experiments were repeated in triplicates. Data were analyzed with SPSS and expressed as the mean ± standard deviation. After confirmation of normality and homoscedasticity, a one-way analysis of variance, Brown–Forsythe and Games-Howell tests were employed to assess the difference between the mean values. Statistical significance was defined as *p* < 0.05.

## Figures and Tables

**Table 1:** Bulk density and textural data from N_2_ physisorption experiments for the SG40TCP60 material used in scaffold manufacture by robocasting and laser micromachining.

Sample	Bulk Density, ρ_Bulk_ (g^−1^ cm^−3^)	S_BET_ (m^2^ g^−1^)	Pore Volume (cm^3^ g^−1^)	Pore Size (nm)	C (BET)
SG40TCP60	1.11 ± 0.01	2.80 ± 1.30	0.03 ± 0.01	13.0 ± 0.1	<0

**Table 2:** Mechanical properties from the uniaxial compression test of the SG40TCP60-RC scaffold and SG40TCP60 composite cylinder; mean values ± standard deviation (*n* = 3 in all cases).

Sample	Young’s Modulus, E (MPa)	Compressive Strength, σ (MPa)	Maximum Compressive Strain, ε (%)
SG40TCP60-RC (Perpendicular)	12.7 ± 5.2	2.00 ± 0.3 *	30 ± 2.2 *
SG40TCP60-RC (Parallel)	18.0 ± 2.7	1.00 ± 0.3	5.3 ± 0.1
SG40TCP60 composite cylinder	56.3 ± 14.5	3.3 ± 0.3	15.2 ± 2.6

* Compressive strength and maximum strain in the perpendicular direction of the RC sample were taken upon the collapse of the layered structures.
